# Transplantation of umbilical cord-derived mesenchymal stem cells into the striata of R6/2 mice: behavioral and neuropathological analysis

**DOI:** 10.1186/scrt341

**Published:** 2013-10-24

**Authors:** Kyle D Fink, Julien Rossignol, Andrew T Crane, Kendra K Davis, Matthew C Bombard, Angela M Bavar, Steven Clerc, Steven A Lowrance, Cheng Song, Laurent Lescaudron, Gary L Dunbar

**Affiliations:** 1 Field Neurosciences Laboratory for Restorative Neurology, Brain Research and Integrative Neuroscience Center, Program in Neuroscience, Central Michigan University, Mount Pleasant, MI 48859, USA; 2 Faculté des Science et des Techniques, Université de Nantes, 44300 Nantes, France; 3 INSERM U1064, ITUN, 44093 Nantes, France; 4 College of Medicine, Central Michigan University, Mount Pleasant, MI 48859, USA; 5 INSERM U791, Laboratoire d’Ingenierie Osteo-Articulaire et Dentaire (LIOAD), 44042 Nantes, France; 6 Field Neurosciences Institute, Saginaw, MI 48604, USA

## Abstract

**Introduction:**

Huntington’s disease (HD) is an autosomal dominant disorder caused by an expanded CAG repeat on the short arm of chromosome 4 resulting in cognitive decline, motor dysfunction, and death, typically occurring 15 to 20 years after the onset of motor symptoms. Neuropathologically, HD is characterized by a specific loss of medium spiny neurons in the caudate and the putamen, as well as subsequent neuronal loss in the cerebral cortex. The transgenic R6/2 mouse model of HD carries the N-terminal fragment of the human HD gene (145 to 155 repeats) and rapidly develops some of the behavioral characteristics that are analogous to the human form of the disease. Mesenchymal stem cells (MSCs) have shown the ability to slow the onset of behavioral and neuropathological deficits following intrastriatal transplantation in rodent models of HD. Use of MSCs derived from umbilical cord (UC) offers an attractive strategy for transplantation as these cells are isolated from a noncontroversial and inexhaustible source and can be harvested at a low cost. Because UC MSCs represent an intermediate link between adult and embryonic tissue, they may hold more pluripotent properties than adult stem cells derived from other sources.

**Methods:**

Mesenchymal stem cells, isolated from the UC of day 15 gestation pups, were transplanted intrastriatally into 5-week-old R6/2 mice at either a low-passage (3 to 8) or high-passage (40 to 50). Mice were tested behaviorally for 6 weeks using the rotarod task, the Morris water maze, and the limb-clasping response. Following behavioral testing, tissue sections were analyzed for UC MSC survival, the immune response to the transplanted cells, and neuropathological changes.

**Results:**

Following transplantation of UC MSCs, R6/2 mice did not display a reduction in motor deficits but there appeared to be transient sparing in a spatial memory task when compared to untreated R6/2 mice. However, R6/2 mice receiving either low- or high-passage UC MSCs displayed significantly less neuropathological deficits, relative to untreated R6/2 mice.

**Conclusions:**

The results from this study demonstrate that UC MSCs hold promise for reducing the neuropathological deficits observed in the R6/2 rodent model of HD.

## Introduction

Huntington’s disease (HD) is an autosomal dominant disorder caused by an expanded and unstable CAG trinucleotide repeat that causes a progressive degeneration of neurons, primarily in the putamen, caudate nucleus and cerebral cortex. The underlying pathology of HD is initiated when the gene that codes for the huntingtin (HTT) protein, located on the short arm of chromosome 4, contains an increased number of CAG repeats [1]. Adult onset HD is characterized by cognitive impairment and psychiatric disturbances, such as irritability, aggressiveness and depression, which precede involuntary motor disturbances [1,2], with death occurring 15 to 20 years later.

The R6/2 mouse model of HD expresses the N-terminal portion of human htt, containing a highly expanded CAG repeat (145 to 155), and develops progressive neurological phenotypes resembling HD [3]. At birth R6/2, mice are indistinguishable from wild-type littermates and have a normal development until six to eight weeks of age when they begin to express the HD phenotype, which consists of neurological signs of stereotypical hindlimb grooming, dyskinesia, irregular gait and motor dysfunction [3,4].

Mesenchymal stem cells (MSC) are multipotent cells derived from adult tissue that are readily available and easily accessed. Previous studies have shown that MSCs can suppress the immune response following transplantation and provide functional efficacy in rodent models of HD. As such, MSCs hold considerable promise as a source for an effective cell therapy [5-7]. However, as MSCs can be obtained from multiple sources, finding the ideal cell source is currently of great interest for optimizing efficacy of stem cell therapies.

As observed previously [8], transplanted bone-marrow-derived MSCs, while capable of reducing behavioral and histological deficits in the R6/2 mouse, did not generate new neurons following transplantation in the mouse striata. Due to this issue, stem cells from other sources, specifically from birth-associated tissues, are gaining interest [5].

The umbilical-cord (UC) is an attractive source of MSCs, as they represent an intermediate link between adult and embryonic tissue, and can be isolated from a noncontroversial source and can be harvested at a low cost [9,10]. Human UC MSCs have also been shown to have a higher harvest rate when compared to bone-marrow-derived cells, making it possible to isolate a substantial number of cells, while limiting the time and number of passages in culture to produce clinically-relevant numbers of cells for transplantation [11,12].

UC stem cells hold advantages over other types of adult stem cells, as it has been shown that UCs do not require human leukocyte antigen (HLA) matching [13,14]. Further, cord-blood is easily cryopreserved, allowing for bio-banking and expansion for future use [10]. It has also been reported that UC MSCs express the pluripotent markers Oct-4, Nanog and Sox-2, albeit at much lower levels than embryonic cells [15]. The expression of pluripotent markers may be due to the nature of the umbilical cord, which lies at an intermediate position between the embryo and the adult organism during development (although the migration of fetal cells between the embryo and adult is not fully understood [16]. It is speculated that some of the fetal cells get ‘trapped’ in the Wharton’s jelly of the umbilical cord [16].

With this potential for pluripotency, many researchers have explored the use of UC MSCs as a cell replacement therapy in neurodegenerative disorders. Several groups have reported that human UC MSCs express neuronal precursor markers and can differentiate into mature neurons *in vitro,* when exposed to the differentiation signals [10,17-21]. In the brain of a Parkinsonian rat, transplanted UC MSCs survived, proliferated and expressed phenotypical markers of dopaminergic neurons [22]. Several studies have also reported behavioral improvements following human UC MSCs transplantations in experimental rat models of middle cerebral artery occlusion [23-25].

The current study tested the efficacy of UC MSCs in the R6/2 mouse model of HD. It was hypothesized that MSCs isolated from the UC would possess the ability to differentiate into neuronal lineages *in vivo* following intra-striatal transplantation. UC MSCs may offer an exciting avenue for transplantation therapies if they are able to exert beneficial factors similar to bone-marrow MSCs, while possessing the ability to differentiate into neuronal lineages.

In order to expand stem cells, specifically MSCs, in sufficient numbers for transplantation, *in vitro* passaging is required, and passaging cells has been shown to alter the properties of these cells [26]. Our previous work suggested that reducing the number of cell passages may increase transplant survivability in rat bone-marrow MSCs and increase their efficacy in reducing behavioral deficits in the 3-nitropropionic acid rat model of HD [27].

The goals of the present experiment were to test: (1) the efficacy of UC MSCs in the R6/2 transgenic mouse model of HD; and (2) how passaging of MSCs *in vitro* alters the functional outcome following transplantation. Behavioral and histological analyses were performed to examine the efficacy of both low-passage (P3 to 8) and high-passage (P40 to 50) UC MSCs transplanted into the striata of R6/2 mice.

## Methods

### *In vitro* cell characterization

The extraction of UC MSCs was performed from day 15 gestation pups of wild-type (WT; C57/BL6 background; Jackson Laboratory, Bay Harbor, ME, USA). Briefly, the placenta was discarded from the distal end of the umbilical cord and the fetus and held above a sterile Petri dish with sterilized forceps. The cells were then pushed out of the umbilical cord using a separate set of sterile forceps. The UC was then diluted in 10 mL MSC medium (Alpha Modified Eagles Medium (αMEM: Invitrogen, Carlsbad, CA, USA) with 10% fetal bovine serum (FBS; Invitrogen), 10% horse serum (HS; Invitrogen), and 5 mg/mL streptomycin and 5 UI/mL penicillin (Sigma; St. Louis, MO)) and collected in a 15 mL Falcon tube and centrifuged at 1,500 rpm for seven minutes at 4°C. The cells were then counted and plated in a 75 cm^2^ flask containing 15 mL of MSC medium.

Following incubation for 48 hours at 37°C, 5% CO_2_, UC MSCs were allowed to attach and non-adherent cells or debris was removed and replaced with fresh MSC medium. When the UC MSCs reached 85% confluency, the cells were passaged. Briefly, the culture medium was aspirated, 0.25% trypsin-ethylenediaminetetraacetic acid (EDTA) solution (Sigma) was added for five minutes to detach the cells, then the trypsin was deactivated with 2 mL of FBS. The trypsin/EDTA solution and FBS containing the cells was collected and centrifuged at 1,500 rpm for seven minutes at 4°C. The supernatant was removed and the pellet was then re-suspended, counted and replated at a density of 8,000 cells/cm^2^ in a new 75 cm^2^ flask (Phenix; Candler, NC) with fresh MSC media.

The low-passage and the high-passage MSCs were analyzed by immunocytochemistry (ICC) and by flow cytometry. Briefly, for ICC, UC MSCs were plated into six-well plates containing poly-L-ornithine coated glass coverslips (25 mm #1; Fisher Scientific; Waltham, MA) and cultured in MSC medium. At 80% confluency, the cells were fixed with 4% paraformaldehyde in 0.1 M PBS at 4°C for ten minutes. To block non-specific binding sites, the coverslips were incubated for one hour at room temperature with 10% normal goat serum (Sigma). Following blocking, the coverslips were incubated in primary antibodies overnight at 4°C. Primary antibodies included CD45 (1/500; Abcam; Cambridge, MA) and SCA-1 (1/500; Abcam). After 24 hours, the coverslips were then rinsed and incubated for one hour at room temperature with the appropriately conjugated secondary antibodies. The secondary antibodies included AlexaFluor488 and AlexaFluor594 (1/300; Invitrogen). The coverslips were then rinsed and incubated in Hoechst 33358 (1/1000; Thermo Scientific; Waltham, MA) for five minutes at room temperature to visualize cell DNA and then mounted onto glass slides using Fluoromount (Sigma). Slides were imaged at 20x using a Zeiss Axiovert 200 M inverted fluorescent microscope.

Flow cytometry analysis followed previously published protocols [28]. Briefly, 200,000 to 300,000 cells were plated into each well of a round-bottom 96-well plate following a passage. The cells were rinsed in 0.1 M PBS containing 1% BSA (Sigma) and 0.1% azide (Sigma) and centrifuged at 2,500 rpm for one minute at 4°C. The cells were then resuspended in 30 μL of primary antibodies for one hour at 4°C. The primary antibodies and dilutions are described above for ICC with the addition of stage specific embryonic antigen 4 (SSEA4, a marker of stem cells, 1/500; Abcam), major histological complex class I (MHC Class I, a receptor for T cell identification; 1/500; Abcam) and MHC Class II, a marker of antigen-presenting cells and lymphocytes. The cells were then rinsed twice and incubated in secondary AlexaFluor488 (1/300; Invitrogen) for one hour at 4°C. The cells were again rinsed twice and fixed using 4% paraformaldehyde for ten minutes on ice. The cells were rinsed and stored at 4°C until analysis was performed using a LSR II (BD Bioscience, San Jose, CA, USA).

RNA isolation was performed from UC MSC cell cultures using a Qiagen RNeasy system (Germantown, MD, USA). All procedures followed the manufacturer’s guidelines. Briefly, two million cells were isolated following passaging and stored at −80°C in 200 μL Trizol (Sigma). Then, 300 μL of buffer RLT was added to the cell pellet and transferred into a gDNA Eliminator tube and centrifuged at 8,000 × g for one minute, at which point 350 μL of 70% ethanol was added to the flow-through and mixed thoroughly. All contents of the flow-through were then added to an RNeasy spin column and centrifuged at 8,000 × g for 30 seconds. The flow-through was discarded, 700 μL of RW1 buffer was added to the RNeasy spin column, and the column was centrifuged at 8,000 × g for 30 seconds. The flow-through was again discarded, and 500 μL of RPE buffer was added to the RNeasy spin column and the column was centrifuged at 8,000 × g for 30 seconds. The RNeasy spin column was placed in a new collection tube, 30 μL of RNase-free water was added to the spin column, and the column was centrifuged at 8,000 × g for one minute. Purified RNA, in the collection tube, was analyzed using a NanoDrop2000 spectrophotometer (ThermoScientific, Asheville, NC, USA) and was stored at −20°C until used for cDNA synthesis. A QuantiTect Reverse Transcription Kit (Qiagen) was used for cDNA synthesis following the manufacturer’s guidelines. Briefly, RNA was incubated at 42°C for two minutes in a genomic DNA elimination buffer. The solution was transferred to a reverse-transcription master mix and incubated at 42°C for 30 minutes and then at 95°C for three minutes to inactivate the reverse transcriptase. The cDNA was stored at −20°C until used in quantitative PCR experiments. Primer used for quantitative PCR was brain-derived neurotrophic factor (BDNF). All values were normalized to the housekeeping gene GAPDH and to a reference sample of tail-tip fibroblasts. Sequences are shown in Table 1.

**Table 1:** Primer sequences used in quantitative RT-PCR analysis

**Primer**	**Sequence**
GAPDH Forward	AAG AGA GGC CCT ATC CCA A
GAPDH Reverse	CAG CGA ACT TTA TTG ATG GTA
BDNF Forward	GAA GAG CTG CTG GAT GAG GAC
BDNF Reverse	TTC AGT TGG CCT TTT GAT ACC

### Animals

All procedures were carried out under the approval of Central Michigan University Animal Care and Use Committee. Male and female R6/2 and WT mice, were housed at 22°C under a 12 hour light/12 hour dark reverse light cycle (lights on at 0900) with *ad libitum* access to food and water. The mice were randomly assigned to one of the following four groups, with the exception of having the groups balanced by gender and genotype: (1) sham-operated (Hanks Balanced Salt Solution; HBSS; Gibco; Grand Island, NY, injection into the striatum) WT mice (WT; n = 17); (2) sham-operated R6/2 (R6/2; n = 12) mice; (3) R6/2 mice transplanted with low-passage UC MSCs (R6/2 UC Low; n = 9); and (4) R6/2 mice transplanted with high-passage UC MSCs (R6/2 UC High; n = 8).

### UC MSC transplantation

At five weeks of age, mice were anesthetized with isoflurane gas and O_2_. The heads of the mice were shaved and cleaned using chlorehexadine (Molnlycke Healthcare, Brunswick, ME, USA). Lidocaine gel (2%, Hi-Tech Pharmacal, Co Inc, Amityville, NY, USA) was placed on the tip of the ear bars prior to placing the mouse in the stereotaxic apparatus. The mice were then placed in the stereotaxic device and the anesthesia was maintained with isoflurane gas and O_2_ for the duration of the surgery. A midline incision was made on the scalp and the skin was retracted, exposing bregma. Two burr holes (0.5 mm) were placed directly over the neostriatum (coordinates relative to bregma: anterior +0.5 mm; lateral + 1.75 mm and −1.75 mm; tooth bar set at −3.3 mm). Prior to transplantation, MSCs at either low passage (P3 to 8) or high passage (P40 to 50) were pre-labeled with Hoechst 33358 (5 μg/mL, Sigma) and resuspended at a density of 200,000 cells per microliter in HBSS. The cells were loaded into a 10 μL Hamilton microsyringe and bilaterally transplanted (−2.5 mm ventral to dura) at a constant rate of 0.33 μL/minute for three minutes. Following the first injection, the syringe was left in place for three minutes, raised 1 mm, and injected a second time, for a total injection volume of 2 μL containing approximately 400,000 cells per hemisphere. After a second three-minute wait period, the microsyringe was withdrawn at a steady rate over a three-minute period. The same procedure was then followed on the opposite hemisphere, the burr holes were sealed with bone wax, and the wound was closed using sterile wound clips (7 mm; CellPoint Scientific, Inc, Gaithersburg, MD, USA). The mice were then placed in a recovery cage until fully mobile, at which point, they were returned to their homecage.

### Behavioral analysis

All mice were tested for baseline behavior at five weeks of age, prior to cell transplantation. Following a one-week resting period after transplantation, the mice were tested weekly for six weeks, on all behavioral tasks, except for the Morris Water Maze (MWM), for which testing started at two weeks post-transplantation. The rotarod task (using the SDI Rotor-Rod; San Diego Instruments, San Diego, CA, USA) was conducted to assess motor coordination. The mice were required to maintain their balance on a 3-cm diameter rotating rod for 60 seconds. The rotarod was set at a constant speed of 10 rpm and each mouse was given three trials per day. If the mouse was incapable of remaining on the rotarod for the full 60 seconds, they fell onto a foam pad placed below the apparatus.

The mice also had their limb-clasping response recorded, which involved suspending them by their tails from a height of 50 cm for 30 seconds. A limb-clasping response was defined as the withdrawal of any limb to the torso for more than one second. Each testing session consisted of three trials, with a clasping score ranging from 0 to 4 (with 0 representing the absence of clasping, 1 representing a withdrawal of any single limb, 2 representing the withdrawal of any two limbs, 3 representing the withdrawal of any three limbs, and 4 representing the withdrawal of all four limbs). The limb-clasping response scores were averaged for each testing session for each animal.

The MWM was used to assess cognitive function through spatial memory. Briefly, the MWM is a 142 cm diameter tank filled with opaque water (30.5 cm deep water mixed with non-toxic white paint). A platform (14 cm diameter) was placed just below (approximately 1 cm) the surface of the water. Prior to the baseline testing week, each mouse was given a cued trial, where the platform was placed in the center of the MWM with a visible flag attached 15 cm overhead. The mice were given four training trials from four starting locations (North, South, East, and West) which shaped them to swim to the escape platform and to ensure that their visual acuity and swimming ability were intact. During each weekly testing period, the location of the hidden platform was altered between the Northwest and Northeast quadrant (with the platform in the center of these quadrants). During baseline and the subsequent testing days, the mice were placed facing the wall of the tank on the center line at the tank’s Southern-most point and given sixty seconds to find the hidden platform. Following a successful trial, the mice were left on the platform for five seconds, removed from the tank, dried, and given a forty-five second inter-trial interval. Mice who did not locate the hidden platform within 60 seconds were guided by hand to the platform and allowed to rest on the platform for five seconds. Mice were given five trials per testing session. The swim speed, distance travelled and latency to escape were tracked and recorded using Viewpoint VideoTrack version 1.75. Measures recorded included latency to find the platform, distance swum, swim speed and the probability of finding the escape platform (number of correct trials/total trials).

### Histological analysis

At the conclusion of behavioral testing, when the mice were 11.5 weeks old, they were deeply anesthetized and overdosed with sodium pentobarbital (delivered i.p.) and transcardially perfused with 0.1 M PBS, followed by 4% paraformaldehyde (diluted in 0.1 M PBS at pH 7.4) to fix the tissue of the animal. The brains were then rapidly removed, suspended in 4% paraformaldehyde for 24 hours at 4°C and then transferred to 30% sucrose in 0.1 M PBS for 48 hours at 4°C. The brains were then flash frozen, using methylbutane and stored at −80°C until they were processed. Coronal sections were cut on a cryostat (Vibrotome UltraPro 5000; Sim Co Ltd, Denizli, Turkey) at 30 μm and were mounted on positive charged microscope slides (Globe Scientific Inc, Paramus, NJ, USA). The tissue was labeled, using previously established free-floating fluorescent staining protocols [27] using antibodies for neuronal nuclei (mouse NeuN; 1/500, Abcam) and glial fibrillary acidic protein (rabbit GFAP; 1/500, Abcam). For immunohistochemical analysis, the tissue was first blocked using 10% normal goat serum in PBS with 0.1% Triton-X (Sigma) for 45 minutes at room temperature. The tissue was then transferred to a well containing the primary antibodies and stored at 4°C overnight. The following day, the tissue was rinsed three times in PBS with 0.1% Triton-X and transferred to a well containing the appropriately conjugated secondary antibodies for one hour at room temperature. Secondary antibodies (1/300; Invitrogen) consisted of anti-rabbit AlexaFluor488 and anti-mouse AlexaFluor594. Images of the fluorescent labels were captured using a Zeiss Axiovert 200 M inverted fluorescent microscope at 20x magnification.

Cytochrome oxidase (CYO) histology was used to provide a terminal measure of metabolic activity in the tissue and these sections were also used for subsequent morphological analyses. Briefly, tissue designated for CYO analysis was submersed in a solution of 800 mg of sucrose (Sigma), 4 mg of cytochrome C (Sigma) and 1 mg of 3, 3'*-*diaminobenzidine (DAB, Vector Laboratories, Burlingame, CA, USA) dissolved in 20 mL of phosphate-buffer for four hours at room temperature. The tissue was then transferred to deionized H_2_O, mounted onto positively charged glass slides and coverslipped using Depex (Electron Microscopy, Hatfield, PA, USA). CYO labelled tissue was scanned using Nikon ScanPro.

All images were analyzed using ImageJ (NIH; Bethesda, MD, USA). Briefly, images of the transplanted cells were captured from five sections of each animal, starting at 0.5 mm anterior to bregma and two sections, approximately 200 μm apart, anterior and posterior to the transplant site. Average intensity of the label, counts of positively labeled cells, as well as percent of co-localization between the transplanted MSCs and NeuN or GFAP, were analyzed in all groups. Densitometric measures of CYO and GFAP were analyzed from images taken in the striatum and the average intensities were normalized to the corpus callosum. Cells were counted as positive if they showed: (1) antibody immunoreactivity within the cell body; (2) the nucleus of that cell was within the counting frame without touching the exclusion lines; and (3) the nucleus of that cell was in focus. For total brain area, five sections (at approximately 100-, 300-, 500-, 700-, and 900 μm anterior to bregma) were traced using ImageJ and the total area was calculated.

### Statistical analysis

All statistical analyses were performed using SPSS v16 with an alpha level equal to 0.05. All behavioral data were analyzed using a repeated measures analysis of variance (ANOVA) to measure changes between genotypes and treatments across weeks. Histological data were analyzed using a multivariate ANOVA. When appropriate, Tukey’s Honestly Significant Difference (Tukey’s HSD) *post hoc* tests were performed.

## Results

### *In vitro* cell characterization

**Figure 1:**
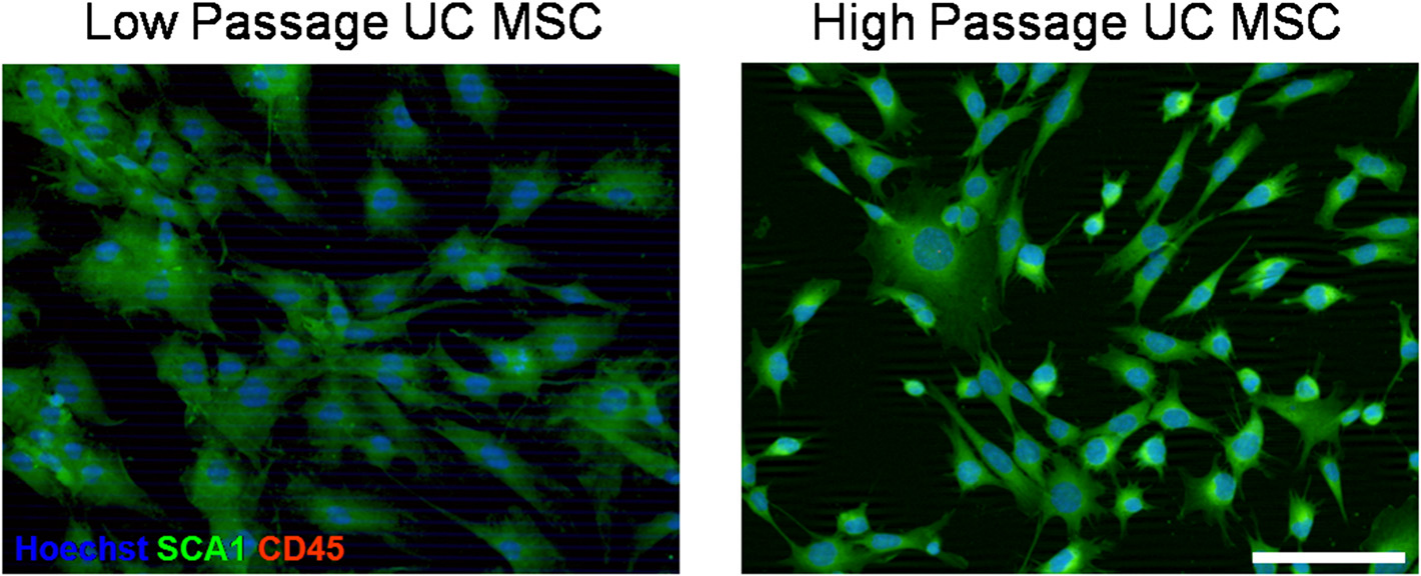
**Immunocytochemistry (ICC) of low- and high-passaged umbilical cord mesenchymal stem cells (UC MSCs).** Low- and high-passaged UC MSCs expressed the MSC marker SCA1, were negative for the hematopoietic stem cell marker CD45 and displayed typical MSC morphology. Scale bar represents 100 μm.

Measures of ICC for both low- and high-passage UC MSCs showed positive expression of SCA1, a marker of mouse MSCs (Figure 1). Flow cytometry revealed that low-passage UC MSCs displayed 26.9% positive expression of SCA-1 and high-passage UC MSCs displayed 30.3% positive expression of SCA-1 (Table 2), contrasting with what was observed previously, where expression of SCA1 increased over passages in bone-marrow derived MSCs. Flow cytometry confirmed ICC analysis revealing that 20.5% of the low-passaged UC MSCs were positive for CD45, a marker of hematopoietic stem cells, while 1.7% of the high-passaged UC MSCs were positive for CD45. The high expression of CD45 at low-passage is most likely due to the fact that the UC MSCs are isolated from tissues containing umbilical cord blood, which contains a high proportion of hematopoietic stem cells. However, these data indicate that CD45 expression is decreasing over passages, either due to prolonged exposure to culture conditions or a selection process occurring during the expansion of cells. Flow cytometry data also revealed that SSEA4, a marker of stem cells, increased from 10.9% in low-passaged UC MSCs to 55.4% in high-passaged UC MSCs. MHC Class I expression remained relatively stable between low- and high-passaged UC MSCs, 2.9% and 1.8%, respectively. However, MHC Class II expression decreased from 10.8% to 1.5% between low- and high-passaged UC MSCs, respectively, suggesting a purification of the MSCs over time.

**Table 2:** Flow cytometry results of low- and high-passaged umbilical cord mesenchymal stem cells (UC MSCs)

	**Passage 5**	**Passage 45**
CD45	20.5	1.7
SCA1	26.9	30.1
SSEA4	10.9	55.4
MHC Class I	2.9	1.8
MHC Class II	10.8	1.5

The number of cells positively expressing SCA1 remained relatively stable as passage number increased. A decrease in the hematopoietic stem cell marker CD45 was observed as cell passage increased. Also, an increase of expression of the stem cell marker SSEA4 between low- and high-passaged cells occurred. Expression of MHC Class I remained relatively stable between low- and high-passaged cells, but a decrease in MHC Class II was observed between low- and high-passaged cells, respectively. MHC, major histological complex; SSEA4, stage specific embryonic antigen 4.

**Figure 2:**
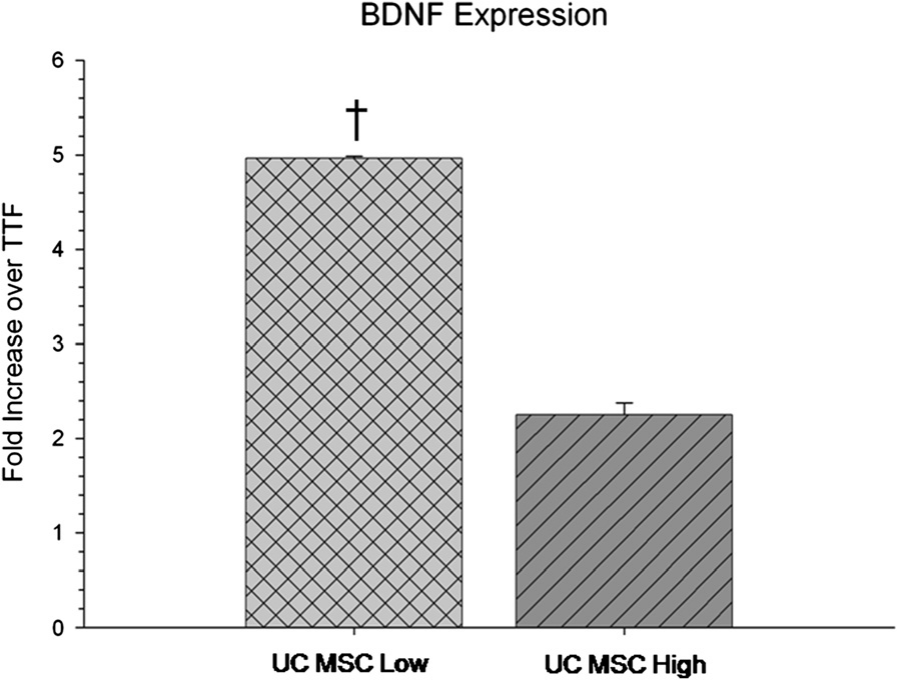
***In vitro *quantitative RT-PCR of BDNF mRNA expression of umbilical cord mesenchymal stem cells (UC MSCs).** A significant decrease in mRNA levels of BDNF was observed in the high-passaged UC MSCs compared to low-passaged UC MSCs. (Note: † significant from high-passage UC MSCs; TTF are control cDNA isolated from mice tail-tip fibroblasts.) Bar graph represents mean value; error bars represents SEM. BDNF, brain-derived neurotrophic factor; SEM, standard error of the mean.

Characterization of UC MSCs through mRNA isolation and RT-PCR revealed significant differences in gene expression of BDNF between low- and high-passaged UC MSCs (*t*(4) = 21.488, *P* <0.001), with low-passaged cells displaying a significantly higher expression of the mRNA for BDNF (Figure 2).

### Behavioral results

**Figure 3:**
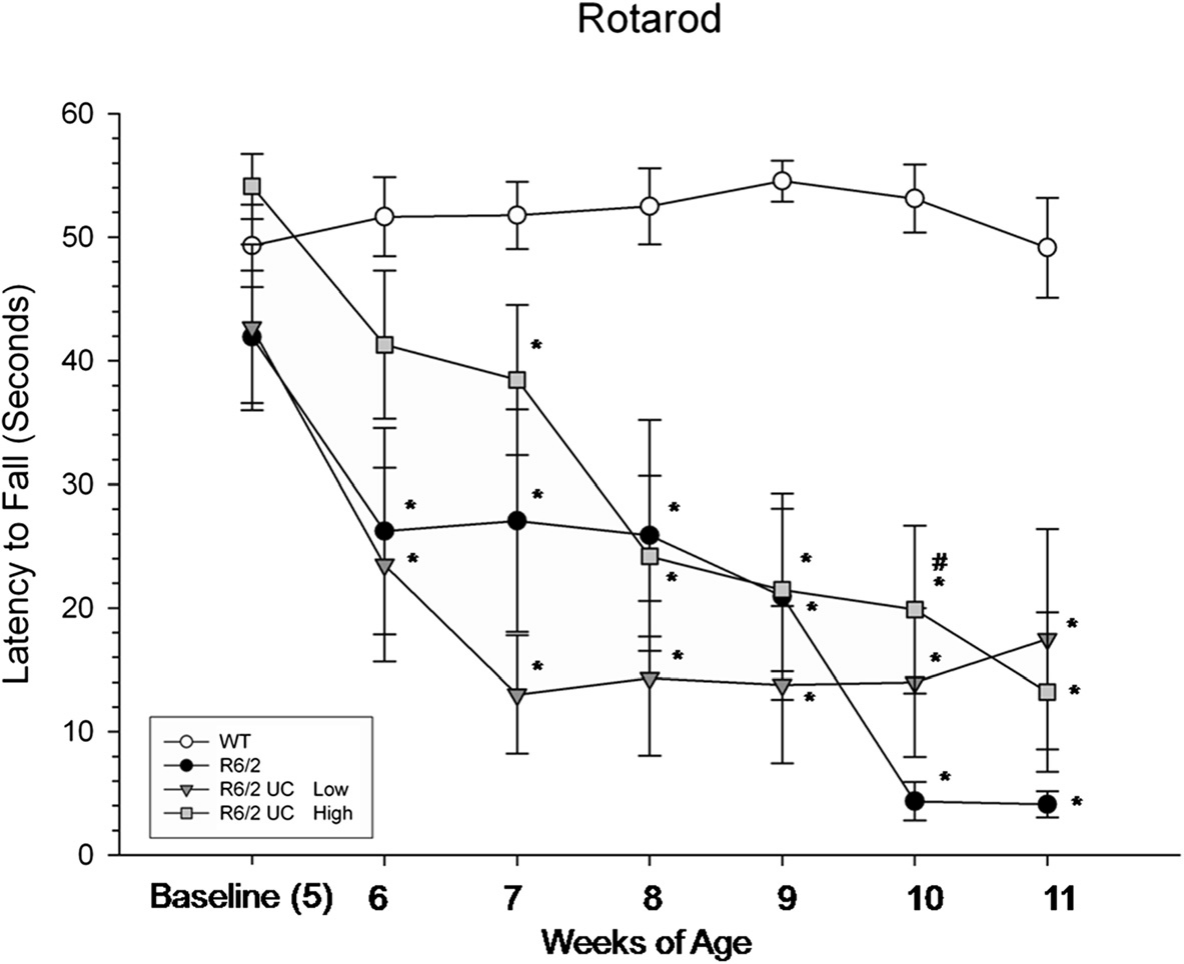
**Motor coordination assessment of R6/2 mice following umbilical cord mesenchymal stem cell (UC MSC) transplantation.** A significant decline in motor coordination was observed in untreated R6/2 mice when compared to WT mice. The R6/2 mice that received transplantation of high-passage UC MSCs displayed significantly longer latencies to fall, compared to untreated R6/2 mice at ten weeks of age. (Note: *significant from WT; ^#^significant from R6/2; ^†^significant from high-passage UC MSCs). Line graph represents mean value; error bars represents SEM. SEM, standard error of the mean; WT, wild type.

In the rotarod task, a repeated-measures ANOVA revealed significant between-group differences for the latency to fall (*F*(3,36) = 13.575, *P* <0.01) (Figure 3). A significant interaction was observed between weeks and group (*F*(18,216) = 5.286, *P* <0.001). Tukey’s HSD analysis revealed significant differences between R6/2 and WT mice starting at six weeks of age and remaining for all testing weeks. Transplantation of low-passage UC MSCs did not confer significant motor benefits, as these animals were similar to R6/2 control mice at all time points. However, significant differences were observed between R6/2 and high-passage UCB MSC mice at 10 weeks of age.

**Figure 4:**
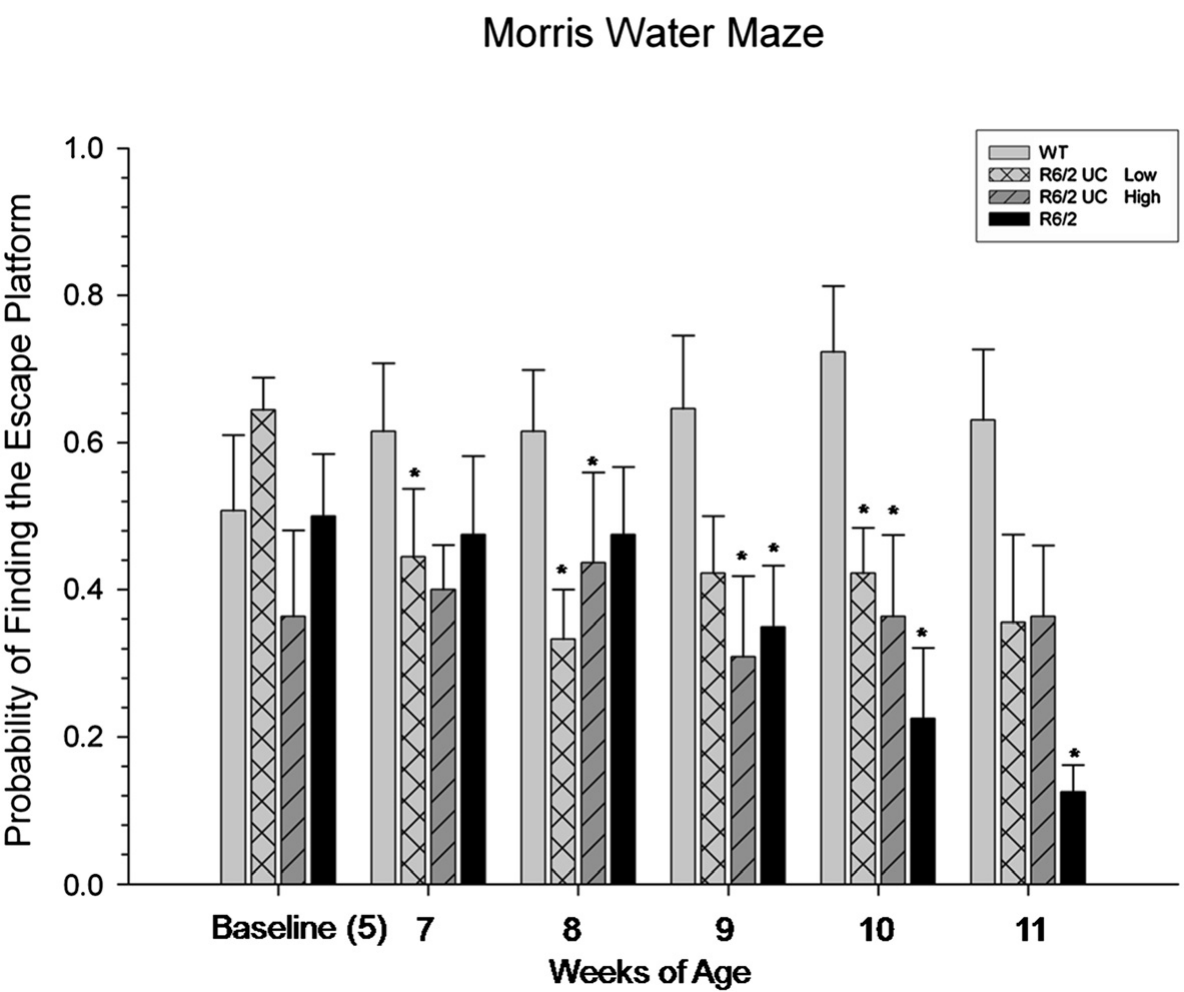
**Spatial memory assessment of R6/2 mice following umbilical cord mesenchymal stem cell (UC MSC) transplantation.** Untreated R6/2 mice displayed significant impairment in spatial memory at 9-, 10- and 11-weeks of age when compared to WT mice. Mice receiving either low- or high-passaged UC MSCs did not display sparing of this spatial memory task. (Note: *significant from WT) Bar graph represents mean value; error bars represents SEM. SEM, stanrard error of the mean; WT, wild type.

Given the high-degree of variability on measures of latency to find the platform and in the distance swum, the probability of finding the hidden platform was used for these analyses. In the MWM task, a repeated-measures ANOVA revealed significant differences between groups for the probability of correctly finding the hidden platform (*F*(3,37) = 4.806, *P* <0.01)(Figure 4). Each trial was scored as ‘correct’ if the mouse was capable of finding the hidden platform in less than sixty seconds and the probability of a ‘correct’ trial was calculated at the end of each testing session (number of correct trials/number of total trials). In this task, untreated R6/2 mice begin to display impairment in spatial memory beginning at nine weeks of age and continuing for the duration of the study. Significant differences were also observed between WT and low-passage UC MSC at seven-, eight- and ten-weeks of age. Significant differences were also observed between WT and high-passage UC MSC at eight-, nine- and ten-weeks of age. Although no significant differences were observed between the UC MSC transplant groups and the untreated R6/2 group, an intermediate effect was observed at 11-weeks of age, with the transplanted groups displaying trends towards (low-passage *P* = 0.13; high-passage *P* = 0.10) having a higher probability of finding the hidden platform than untreated R6/2 mice. It is important to note that the R6/2 mice did not display motor deficits in swimming ability and their swim speeds were similar to WT mice throughout all testing periods. However, their ability to locate the hidden platform within the 60-second trial period was significantly different than WT mice, and this deficit was mitigated somewhat by the UC MSC transplants.

**Figure 5:**
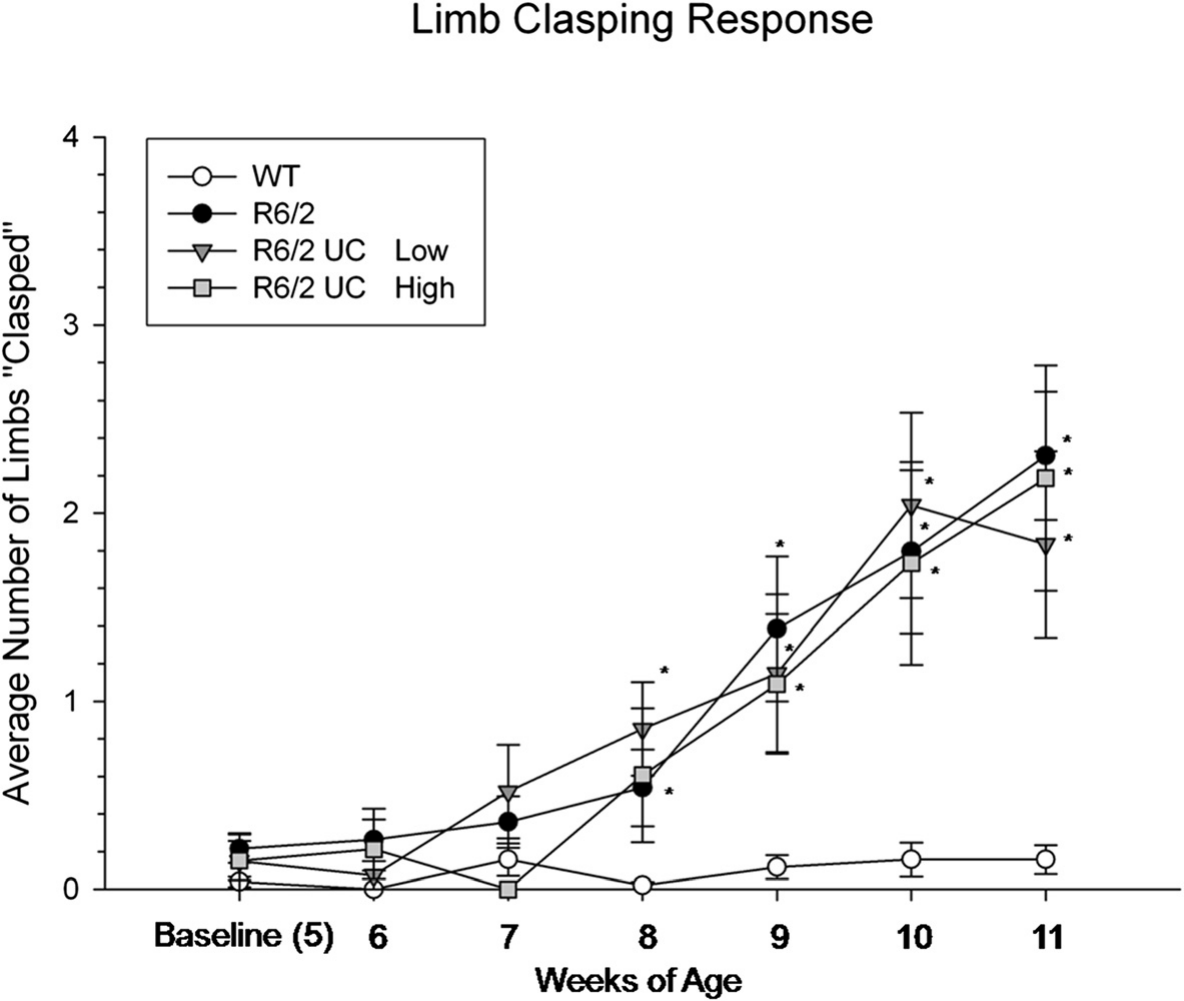
**Limb-clasping of R6/2 mice following umbilical cord mesenchymal stem cell (UC MSC) transplantation.** Untreated R6/2 mice had significantly more limb-clasping responses than did WT mice, starting at eight-weeks of age, with this impairment continuing for the duration of the study. Mice receiving either low- or high-passaged UC MSCs were similar to untreated R6/2 mice. (Note: *significant from WT; ^#^significant from R6/2; ^†^significant from high-passage BM MSCs). Line graph represents mean value; error bars represents SEM. BM, bone marrow; SEM, standard error of the mean; WT, wild type.

A repeated-measures ANOVA revealed significant differences between groups for limb-clasping (*F*(3,42) = 8.259, *P* <0.001), and a significant interaction was observed between weeks and group (*F*(18,252) = 4.845, *P* <0.001)(Figure 5). This phenotypic difference observed in R6/2 mice began at eight weeks of age. No effect of UC MSC transplantation was observed, as all transplanted animals had similar scores, relative to untreated R6/2 mice. Contrary to what has been previously observed in our lab using bone-marrow MSCs, a reduction of the limb-clasping was not observed in the animals that received transplantation of UC MSCs.

### Histological results

**Figure 6:**
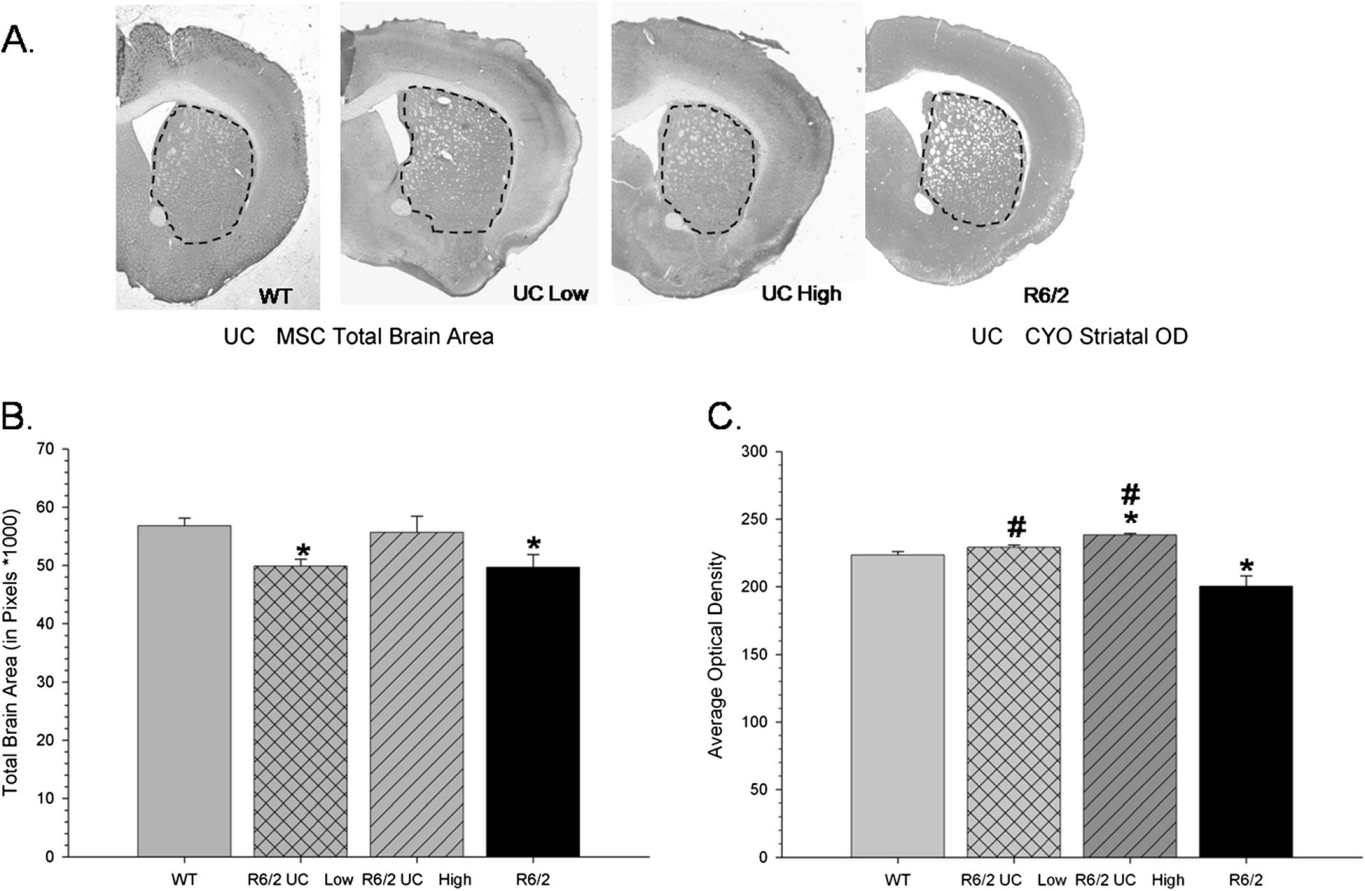
**Measures of brain area and evidence of integrity of the metabolic tissue in the striata of mice receiving umbilical cord mesenchymal stem cell transplantations.** Gross morphology of the brain near the area of transplantation can be visualized with cytochrome oxidase labeling **(A)**. Untreated R6/2 mice and mice that received transplantation of low-passaged UCMSCs had a significant decrease in total brain area when compared to WT mice **(B)**. Optical densitometric measures of cytochrome oxidase in the striata (outlined in dashed line) revealed significantly less metabolic activity of striatal tissue in untreated R6/2 mice, when compared to WT mice at the time of necropsy **(C)**. R6/2 mice that received transplantation of low- or high-passaged UC MSCs had significantly higher levels of metabolic activity in striatal tissue than did untreated R6/2 mice. (Note: *significant from WT; ^#^significant from R6/2). Line graph represents mean value; error bars represent SEM. SEM, standard error of the mean; UCMSCs, umbilical cord mesenchymal stem cells; WT, wild type.

Following perfusion and histochemical analysis of CYO labelled tissue, a one-way ANOVA revealed significant between group differences in the area of the whole brain from the sections analyzed (*F*(3,109) = 4.414, *P* <0.01)(Figure 6A and B). Tukey’s HSD analysis revealed that the untreated R6/2 mice had significantly smaller brain areas than did WT mice, suggesting generalized brain atrophy. The mice that received transplantation of high-passaged UC MSCs did not significantly differ from WT mice on measures of brain area, an observation that was not detected in the low-passaged UC MSC transplanted mice. Optical densitometric measures of metabolic activity of striatal tissue revealed a significant between-group difference (*F*(3,109) = 7.846, *P* <0.001) (Figure 6C). Tukey’s HSD tests revealed that R6/2 mice had significantly less CYO labeling than WT and R6/2 mice transplanted with low- or high-passage UC MSCs. While transplantation of low-passage UC MSCs did not protect against the loss of overall brain area, the low-passaged UC MSCs were able to reduce the loss of metabolic activity in striatal tissue, compared to untreated R6/2 mice as determined by optical densitometric measures. Transplantation of high-passaged UC MSCs preserved significantly more metabolic activity in striatal tissue than what was observed in either untreated R6/2 mice or, interestingly, WT mice.

**Figure 7:**
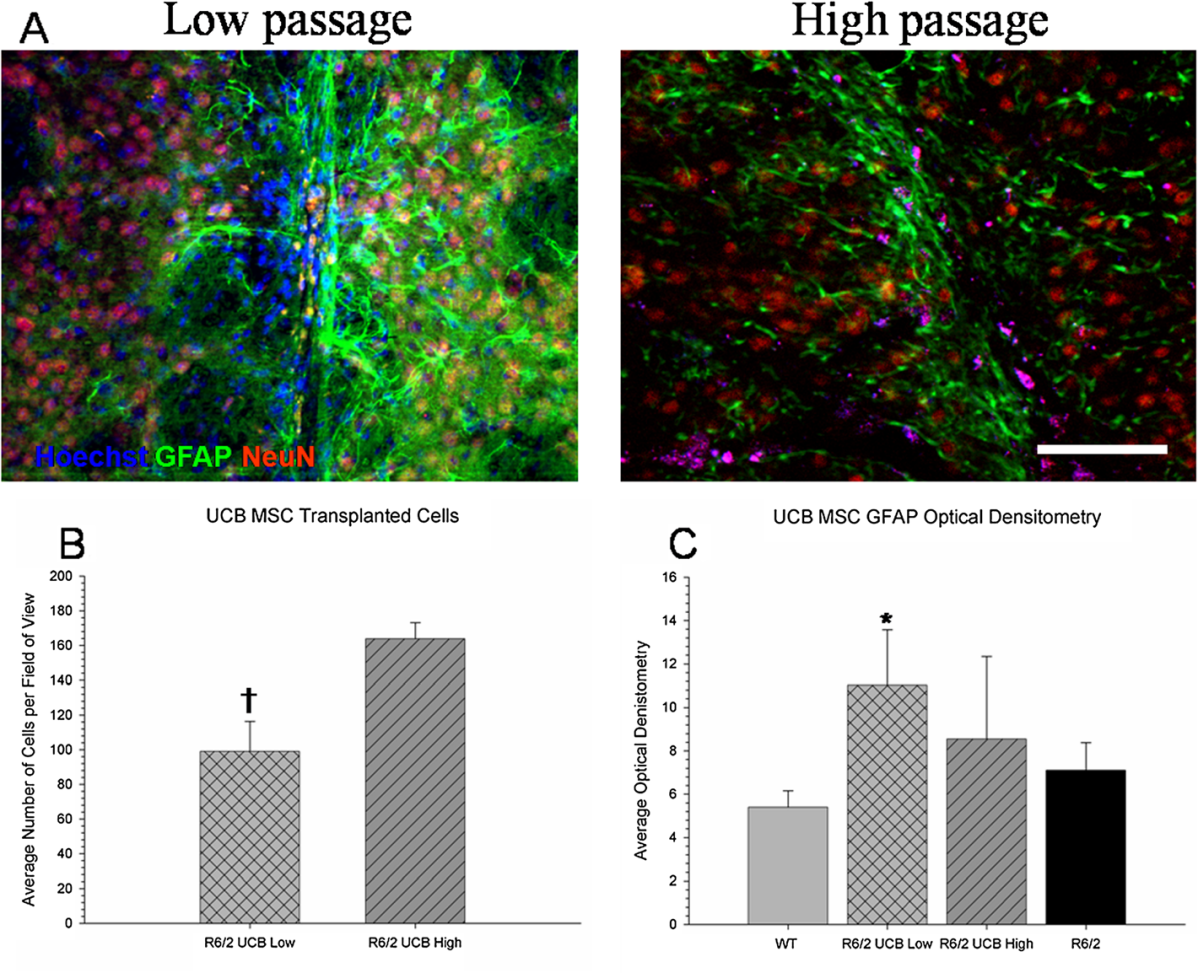
**Immunohistochemical analysis of the transplanted umbilical cord mesenchymal stem cells (UC MSCs).** No neuronal or glial differentiation was observed from the transplanted UC MSCs as seen by the lack of co-localization between the UC MSCs (blue) with NeuN (red) or GFAP (green), respectively **(A)**. A decreased significant difference in the amount of surviving transplanted cells was observed in low-passage UC MSCs, when compared to high-passage UC MSCs **(B)**. A significant increase in GFAP optical densitometry around the transplant site was observed in the R6/2 mice that received transplantation of low-passage UC MSCs **(C)**. GFAP, glial fibrillary acidic protein; NeuN, neuronal nuclei.

Student’s *t-*test revealed a significant difference in the number of surviving UC MSCs between the low- and high-passaged groups at six weeks following transplantation (*t*(55) = 3.104, *P* <0.05) (Figure 7B). It was observed that mice receiving transplantation of high-passage UC MSCs had significantly more surviving cells at six weeks post-transplantation than mice receiving low-passaged UC MSCs. This may explain why mice receiving high-passage UC MSCs tended to show slightly more behavioral sparing, less brain area loss, and significantly higher CYO measures of metabolic activity in the striatum than mice receiving low-passage UC MSCs, in spite of the fact that low-passaged UC MSCs had a higher expression of BDNF *in vitro.* Student’s *t-*test revealed no significant between-group differences in measures of optical densitometry of GFAP from the area around the transplant site (*t*(55) = 2.265, *P* >0.05)(Figure 7C). These data suggest that the deficits observed in the R6/2 mice are probably not due to astrocyte activation, and any behavioral or histological sparing that was observed is unlikely to be due to either the up- or down-regulation of astrocytes in the brain. It also demonstrates that the number of passages a cell undergoes does not significantly modulate astrocyte activation following intra-striatal transplantation.

In contrast to what was hypothesized, little to no co-localization of the transplanted UC MSCs with the mature neuronal marker NeuN was revealed, suggesting that these cells did not differentiate into mature neurons *in vivo* following transplantation. This is in contrast to what has been previously reported following transplantation of UC MSCs in the brain [22-25], but similar to what has been observed following bone-marrow MSC transplantation [7,27,29,30].

## Discussion

The four main findings of this study were: (1) transplantation of UC MSCs into the striata of R6/2 mice provided transient behavioral sparing compared to mice that did not receive stem cell transplants; (2) transplantation of high-passage UC MSCs resulted in a significant reduction in neuropathology, albeit providing only transient behavioral sparing; (3) the number of times the cells were passaged significantly altered the number of surviving cells and the astrocyte activation to the transplant 6.5 weeks following the transplant; and 4) while the UC MSCs did express the mRNA for BDNF *in vitro*, this alone was not able to provide significant behavioral sparing, suggesting that the transient reduction in behavioral deficits following transplantation of UC MSCs is not solely due to BDNF expression but likely due to a release of several trophic factors and immunomodulating cytokines [27].

Data from this study demonstrate that transplantation of UC MSCs, while providing transient behavioral sparing, did not provide robust reductions of deficits to the extent of those previously observed in our lab following transplantations of bone-marrow-derived MSCs [7,29,30]. Transplantation of high-passaged UC MSCs were capable of delaying the motor deficits compared to untreated R6/2 mice when the mice were 6- and 10-weeks of age. An intermediate effect was observed in mice receiving UC MSC transplants in the MWM at 11-weeks of age.

While long-term behavioral sparing was not observed following intrastriatal UC MSC transplantation in these mice, significant reductions in neuropathology, in terms of preserved optical densitometric measures of CYO labeling in the striatum of R6/2 mice that received either low- or high-passaged UC MSCs. In addition, mice that received high-passaged UC MSCs also did not show overall brain atrophy when compared to untreated R6/2 mice. The trend for reduced neuropathological deficits observed in the high-passaged group, relative to the low-passaged group may, in part, be due to the number of surviving cells, as well as a reduced immune response to those cells. It was observed that there were significantly more surviving cells in the high-passaged group and that there was an increase in the optical densitometric measures of GFAP in the low-passaged group, when compared to the high-passaged group, suggesting that the low-passaged group may be subjected to a greater immune response, resulting in fewer surviving cells.

Because our results suggested that high-passaged UC MSCs tend to provide greater behavioral and neuropathological sparing than low-passaged UC MSCs, we were surprised that the low-passaged UC MSCs displayed a higher expression of mRNA for BDNF *in vitro.* This suggests that the mechanism underlying MSC-mediated recovery is not solely dependent on BDNF, but probably involves a host of other trophic and immunomodulatory factors. Surprisingly, mRNA expression of BDNF in UC MSCs are in contrast to what was previously observed in our study with bone-marrow MSCs, whereby murine MSCs that have been maintained in culture for more than 40 passages had higher expression of BDNF mRNA than those that had been maintained in culture for less than eight passages. While it has been suggested that deficits in BDNF production play a causal role in the progression of HD [31] and the increasing levels of BDNF may underscore behavioral sparing following MSC transplantation into rodent models of HD, the immune response to these cells also needs to be closely examined, as has been observed in other studies [7,8,27,29].

A main goal of utilizing MSCs isolated from the UC was that these cells may possess greater levels of pluripotency than other adult MSCs, due to their intermediate developmental status between the fetus and the adult. However, in our lab, MSCs isolated from the UC, while displaying typical MSC morphology and protein expression, did not express markers of pluripotency and were unable to differentiate into neuronal phenotypes *in vivo* following intrastriatal transplantation. While we were unable to discern umbilical cord blood from Wharton’s jelly during cell isolation, the cells isolated did display characteristics of MSCs and were able to confer modest behavioral and anatomical sparing.

## Conclusions

The results from this study demonstrate that UC MSCs may hold significant therapeutic value for reducing the neuropathological changes observed in the R6/2 rodent model of HD. While the cells cultured in our standard MSC medium did not express markers of pluripotency, changing the protocols for cell extraction from the umbilical cord [17,20] or exposing the UC MSCs to different culture conditions [19] may increase the therapeutic efficacy of these cells. Stem cells from perinatal sources are gaining interest for clinical applications as these cells carry fewer ethical concerns and are easily isolated after birth. Our findings highlight the use of mesenchymal stem cells isolated from the umbilical cord, but other sources of extra-embryonic sources are gaining interest as well, such as the placenta [32]. In addition, new sources of pluripotent stem cells are being considered for transplantation therapies in neurodegenerative diseases [33,34], and extra-embryonic cells may provide a viable starting cell to generate therapeutically relevant induced pluripotent stem cells.

## Abbreviations

ANOVA: Analysis of variance; BDNF: Brain-derived neurotrophic factor; CYO: Cytochrome oxidase; EDTA: Ethylenediaminetetraacetic acid; FBS: Fetal bovine serum; GFAP: Glial fibrillary acid protein; HBSS: Hank’s balanced salt solution; HD: Huntington’s disease; HLA: Human leukocyte antigen; HSD: Honestly significant difference; HTT: Huntingtin; ICC: Immunocytochemistry; MHC: Major histological complex; MSC: Mesenchymal stem cell; MWM: Morris water maze; NeuN: Neuronal nuclei; PBS: Phosphate-buffered saline; PCR: Polymerase chain reaction; SSEA4: Stage specific embryonic antigen 4; UC: Umbilical-cord; WT: Wild-type.

## Competing interests

The authors declare that they have no competing interests.

## Authors’ contributions

KF conducted behavioral testing, histological and RT-PCR analysis, performed statistical analysis, participated in the design of the study and drafted the manuscript. JR conducted behavioral testing, histological analysis, participated in the design of the study and coordination and helped to draft the manuscript. AC conducted histological and RT-PCR analysis and helped to draft the manuscript. KD conducted behavioral testing and histological analysis. MB conducted behavioral testing, histological analysis and participated in the design of the study. AB conducted behavioral testing and histological analysis. SC conducted behavioral testing and histological analysis. SL conducted behavioral testing and helped to draft the manuscript. CS participated in genotyping the animals. LL participated in the design and coordination and helped to draft the manuscript. GD conceived of the study, and participated in the design and coordination and performed the final proof of the manuscript. All authors read and approved the final manuscript.
